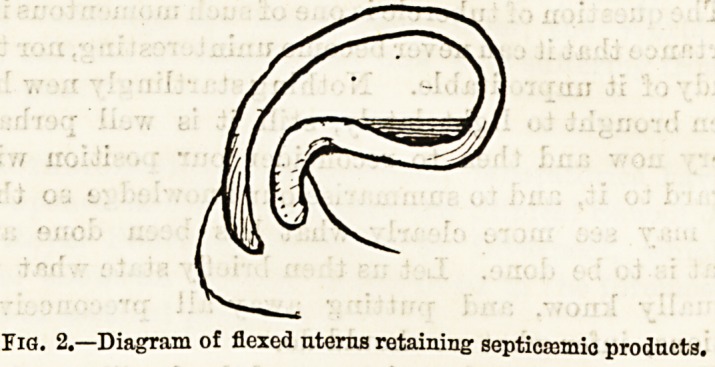# Intra-Uterine Syringing in Puerperal Septicæmia

**Published:** 1894-05-19

**Authors:** Norman Porritt

**Affiliations:** Surgeon, Huddersfield Infirmary


### INTRA-UTERINE SYRINGING IN PUERPERAL SEPTICÆMIA.

By Norman Porritt, M.R.C.S., L.R.C.P., Surgeon, Huddersfield Infirmary.

Given a case of puerperal septicæmia the desirability of intra-uterine irrigation will have to be considered. When careful examination fails to discover portions of retained placenta or clots the uterine catheter should be used to wash away decomposing *débris* beyond the reach of the finger, and when putrefying substance has been found and removed the catheter will complete the work of the finger.

A good intra-uterine catheter is broad and blunt ended; long enough to reach beyond the external parts when the end is at the top of the uterus, and is provided with an arrangement to ensure the unimpeded return of the injected fluid. Budin's catheter, a hollow instrument of celluloid or glass, half an inch

Fig. 1.—Budin's catheter for intra-uterine syringing. Made by Down.

broad and at least twelve inches long, with a deep groove along the whole of its convex side best fulfils the conditions laid down. The uterine cervix is patent enough to admit such an instrument; its breadth and bluntness are a guarantee that the soft uterine tissues cannot be punctured; whilst the grooved channel provides a never closing exit for the intra-uterine contents. If there is difficulty in introducing the instrument it is due to flexion of the enlarged uterus. Some practitioners keep several instruments of different degrees of curvature to meet this difficulty; but with a long instrument it is usually possible to gently manipulate the point until the direction of the uterine cavity is found and the catheter passes. The escape of a quantity of pus down the groove is sometimes the indication that the catheter is finding its way into position, the fundus of the uterus having been converted by the flexion into a closed pus-containing cavity. Not the least valuable of the benefits of intra-uterine syringing is the straightening of the uterus, and the consequent opening of the pent house of septicæmic purulence. (Fig. 2.)

Fig. 2.—Diagram of flexed uterus retaining septicæmic products.

It is essential that the whole length of the uterine cavity should be washed, and this can best be done by an instrument of the length indicated. For it is inconvenient to use an instrument of which the lower end is in the vagina; and, on the other hand, if the distal end reaches just through the cervical canal there is the danger of washing detritus higher up into the cavity instead of by a backward current sending it into the vagina. The celluloid instruments have the advantage that they are softened by hot water and can then be bent to the required curvature. Moreover, they are easily cleaned and made aseptic.

Diluted Condy's fluid, a solution of salufer (ten grains to the pint), or of carbolic acid (two fluid drams of the liquefied acid to the pint) may be used. The penetrating and antiseptic properties of carbolic acid are unsurpassed, and if the acid be quite dissolved in the hot water used it is free from disadvantages.

Undissolved globules of the strong acid may settle at the bottom of the solution, and may at the very last find their way into the uterus, and, causing a local inflammation, may add to the dangers and assist the spread of the disease. If the acid be previously mixed with its own volume of glycerine, or if the glycerin: acidi carbolici be used in making the solution, there will be no difficulty in effecting complete solution.

The solution may be thrown into the uterus by a Higginson's syringe attached to the catheter, or by an irrigation glass or tin. For safety and convenience the irrigator is preferable. A folded blanket or sheet having been passed under the patient, a bidette or bed-slipper is placed under her as she lies upon her back near the left edge of the bed. The irrigation tin having been filled and given in charge of the nurse, who also has close at hand a jugful of solution to replenish the tin as the injection proceeds, the attendant, standing on the left side of the patient, takes the well-lubricated catheter in the right hand, and passing it out of sight under the bed-clothes, allows the fluid to run into the bidette, until the liquid comes through quite warm. With the left forefinger he then guides it into the vagina, flushes that out on all sides, and passing the finger to the os uteri guides the catheter along it into the patulous opening; and then with the utmost gentleness he sends on the instrument, at the same time that he depresses the outer end to the perineum. The instrument having been passed, the finger is removed from the vagina, the catheter is held in position and the irrigation proceeds. From three to four pints of liquid may be used with an ordinary-sized slipper without wetting the bed.

It is usually recommended to place the patient on her side near the edge of the bed, but this involves needless change of position and unnecessary exposure. Sometimes such change of position is a great tax upon the powers of the patient; and bearing in mind the predisposition of these cases to thrombosis the fear that the movement may dislodge clots in the vessels is not imaginary, as was proved by a fatal case occurring in this manner in the practice of the writer. Moreover, the dorsal position is more pleasant for the patient. All manipulations are conducted out of sight beneath the bedclothes, the nurse places the patient ready for the catheter to be used, there is no chilling of the surface, except by mismanagement the bed is not wetted, and the patient's objections to the slight discomfort to which she is subjected are very different from the sometimes active opposition to the night and morning change to the lateral position in a situation convenient for the manipulations of the attendant. Again, the catheter passes as readily with the patient in the dorsal, as in the left lateral position; whilst the time spent in preparing and placing the patient is considerably less—an important consideration to a busy practitioner.

The strong, steady current from an irrigator is just as efficient as the intermittent spurts of a Higginson's syringe. In using it the attendant has both hands free, and so long as the nurse keeps the tin replenished air cannot get into the uterus. If the valved end of the Higginson's syringe slips out of the dish, air may enter the apparatus, and the catheter must then be removed and reintroduced after the air has been expelled and the apparatus refilled with fluid, unless the attendant desires to inject air with the fluid he sends into the uterus.

In all the manipulations there should be no approach to either roughness or hurry. The attendant should have ever before him the danger of adding to the existing local inflammation, every increase of which favours the conversion of a local septic nidus into a fatal widespread affection.

The uterine syringing should be repeated twice daily so long as the temperature continues above 101 degrees, or so long as pus or decidual remnants continue to be washed away, even though the temperature falls below 101 degrees. The attendant should be on the look-out for complications, and should not mistake a rise of temperature due to a pneumonia, a phlebitis, a pleurisy, or a local abscess for that of a continuance of the septicæmia.

## Figures and Tables

**Fig. 1. f1:**
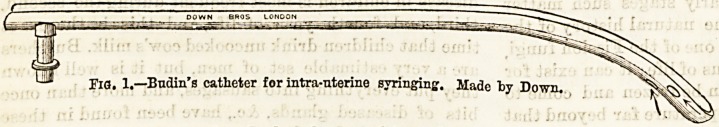


**Fig. 2. f2:**